# Diversity and Distribution of Volatile Secondary Metabolites Throughout *Bacillus subtilis* Isolates

**DOI:** 10.3389/fmicb.2020.00559

**Published:** 2020-04-08

**Authors:** Marco Kai

**Affiliations:** Institute for Biological Sciences, University of Rostock, Rostock, Germany

**Keywords:** volatiles, VOCs, secondary metabolites, *Bacillus subtilis*, GC/MS, GC/EI-MS, identification

## Abstract

*Bacillus subtilis* releases a broad range of volatile secondary metabolites, which are considered as long- and short distance infochemical signals mediating inter- and intra-specific processes. In addition, they often show antimicrobial or antifungal activities. This review attempts to summarize yet known volatile secondary metabolites produced and emitted by *Bacillus subtilis* isolates focusing on the structural diversity and distribution patterns. Using *in vitro* volatile-collection systems, 26 strains of *B. subtilis* isolated from different habitats were found to produce in total 231 volatile secondary metabolites. These volatile secondary metabolites comprised mainly hydrocarbons, ketones, alcohols, aldehydes, ester, acids, aromatics, sulfur- and nitrogen-containing compounds. Reviewed data revealed to a great extent isolate-specific emission patterns. The production and release of several volatile bioactive compounds was retained in isolates of the species *B. subtilis*, while volatiles without a described function seemed to be isolate-specifically produced. Detailed analysis, however, also indicated that the original data were strongly influenced by insufficient descriptions of the bacterial isolates, heterogeneous and poorly documented culture conditions as well as sampling techniques and inadequate compound identification. In order to get deeper insight into the nature, diversity, and ecological function of volatile secondary metabolites produced by *B. subtilis*, it will be necessary to follow well-documented workflows and fulfill state-of-the-art standards to unambiguously identify the volatile metabolites. Future research should consider the dynamic of a bacterial culture leading to differences in cell morphology and cell development. Single cell investigations could help to attribute certain volatile metabolites to defined cell forms and developmental stages.

## Introduction

*Bacillus subtilis* is a rod-shaped, aerobic, endospore forming Gram-positive bacterium that colonizes the soil and often occurs plant-associated. Especially the rhizosphere was found to be a frequent natural habitat (Kunst et al., 1997). This habitat is also highly populated with other bacteria, fungi, and proto- and metazoa. In order to cope with this competitive environment, *B. subtilis* is able to release a large number of metabolites, which can modify the performance of co-habitants from the neighborhood (Romero et al., 2007; Sansinenea and Ortiz, 2011; Harwood et al., 2018). Many of these metabolites are classified as secondary metabolites, since there is no essential need for them in growth, development, or reproduction of the organisms. Nevertheless, their absence can ultimately cause damage or alter the organism or population, since secondary metabolites function as communication signals, antibiotics or siderophores (Vining, 1990; Davies, 2006; Yim et al., 2007; Ratcliff and Denison, 2011; Sansinenea and Ortiz, 2011). Due to their physico-chemical properties, secondary metabolites are grouped into non-volatile and volatile compounds. Non-volatile secondary metabolites of *B. subtilis* comprises lipopeptides (classes of surfactin, iturin, and fengycin), polyketides and non-ribosomal peptides. These compounds were excellently described in the past (Kakinuma et al., 1969; Peypoux et al., 1981; Hofemeister et al., 2004; Stein, 2005; Schneider et al., 2007; Sansinenea and Ortiz, 2011; Harwood et al., 2018; Caulier et al., 2019). Although no strain is able to produce a complete spectrum of non-volatile secondary metabolites, their release is found throughout the majority of investigated *B. subtilis* isolates (Stein, 2005).

Volatility improves the efficacy of a large number of secondary metabolites. High vapor pressure enables compounds with low molecular weight to act in the close vicinity of the producing organism but also to travel over longer distances even in soil (Rasmann et al., 2005; Effmert et al., 2012; Schulz-Bohm et al., 2018). Bacteria in general release a high diversity of volatile secondary metabolites including hydrocarbons, ketones, alcohols, sulfur- and nitrogen containing compounds, terpenes, and others (Schulz and Dickschat, 2007; Lemfack et al., 2014, 2018). These metabolites are primarily considered as long- and short distance infochemicals mediating inter- and intra-specific interactions, but they also act as antimicrobial or antifungal agents (Kai et al., 2009; Schmidt et al., 2017; Schulz-Bohm et al., 2017). Actinomycetes, for instance, produce the earthy-smelling terpene geosmin (Gerber and Lechevalier, 1965), which can be used by Drosophila flies to detect toxic bacteria (Stensmyr et al., 2012). Another well-known bacterial volatile is the multifunctional dimethyl disulfide (DMDS) produced by a wide range of bacteria including *Bacillus* spp., *Burkholderia* spp., *Pseudomonas* spp., *Serratia* spp. and *Streptomyces* spp. (Lemfack et al., 2018). While in insects DMDS acted insecticidal by hindering the electron transport via inhibition of cytochrome oxidase and blockage of potassium channels (Dugravot et al., 2003; Gautier et al., 2008), plants can incorporate DMDS into plant proteins leading to plant growth promotion (Meldau et al., 2013). In high doses, however, DMDS inhibited plant growth by a mechanism that is not yet known (Kai et al., 2010).

Fiddaman and Rossall (1993) reported that a *B. subtilis* strain releases volatiles with antifungal properties. Ten years later, Ryu et al. (2003, 2004) found out that volatile metabolites emitted by the *Bacillus subtilis* isolate GB03 significantly promoted plant growth and induced systemic resistance in *Arabidopsis thaliana*. Although the isolate GB03 was later newly classified as *Bacillus amyloliquefaciens* GB03 (Choi et al., 2014), these impressive results encouraged research groups to consider *B. subtilis* as a producer of bioactive volatiles.

The latest review on secondary metabolites of *B. subtilis* was published by Caulier et al. (2019). They included non-volatile and volatile compounds, but mainly focused on substances with antimicrobial properties. Yet, there is no comprehensive overview of only volatile secondary metabolites of *B. subtilis* regardless of their specificity or general properties and biological and ecological role. Therefore, this review attempts to summarize known volatile secondary metabolites of known *B. subtilis* isolates focusing on structural diversity and distribution in the various isolates.

## Volatile Secondary Metabolites Emitted by *Bacillus Subtilis*

To get a comprehensive insight into the vast amount of volatile secondary metabolites emitted by *B. subtilis* I searched for documented *B. subtilis* isolates in the literature and in the database mVOC (Lemfack et al., 2018). Studies of single volatiles (e.g., acetoin and isoprene) were not considered in this review. The search revealed 26 *B. subtilis* strains and their corresponding volatile-profiles (Table 1) investigated in twenty studies. This included mainly isolates from different soil habitats (tobacco fields, rhizosphere of rice, cucumber or cabbage, greenhouse soil) and isolates from food sources (bean curd, oranges, and cooked food). For some isolates, the origin was not described or not known. Although several hundred wild-type strains of *B. subtilis* have been analyzed for non-volatile metabolites (Stein, 2005), the present review will give a first overview of volatile secondary metabolites.

**TABLE 1:** Investigated *Bacillus subtilis* isolates.

***Bacillus subtilis* isolate**	**Habitate**	**Medium used for VOC-collection**	**Collection**	**Analysis**	**Identification**	**References**
*B. subtilis* ATCC 6051	Unknown (Zeigler et al., 2008)	Tryptic soy broth (TSB)	Static system – culture shaked at 30°C	SPME – PDMS at day 5 of cultivation	Medium comparison – Wiley 138K Mass Spectral Database, authentic standards for most chemicals	Robacker et al. (1998)
*B. subtilis* 168	Mutant of the *Bacillus subtilis* type strain Marburg (Burkholder and Giles, 1947)	Murashige–Skoog (MS) medium containing 1.5% (w/v) agar, 1.5% (w/v) Suc, and 0.4% (w/v) TSA	Dynamic open air stream system – Plates in a Teflon framed chamber at 28°C	Trapping – Super-Q adsorbent traps at 48 h of cultivation – intervals of 24 h over 6 days	Medium comparison – EPA/NIST library, authentic standards	Ryu et al. (2004)
		Lysogeny broth (LB)	Static system – 7 ml in culture a 20 ml vial at 37°C	SPME – DVB/CAR/PDMS at 24 h of cultivation	Medium comparison – NIST/EPA/NIH Mass Spectral Database (NIST 05)	Li et al. (2015)
*B. subtilis* 7, 8 and 9	Tobacco fields	Beef extract peptone broth (BEPB)	Static system – 9 ml in a 15 ml vial at 37°C	SPME – coating not defined at 24 h of cultivation	medium comparison – Wiley 138 and NBS 75 k library (similarity index > 850)	Gu et al. (2007)
*B. subtilis* B2g	Rhizosphere of *Brassica napus* (Berg et al., 2002)	Nutrient agar (NA)	Dynamic open air stream system – plates in a glass chamber at 30°C	Trapping – Super-Q adsorbent traps at 48 h of cultivation for 24 h	Medium comparison – no compound detected	Kai et al. (2007)
*B. subtilis* JA	Soil	LB	Dynamic open air stream system – 100 ml culture in 250 ml flasks	Trapping – charcoal adsorbent traps	Medium comparison – NIST/EPA/NIH Mass Spectral Library (Software Version 2.0)	Chen et al. (2008)
*B. subtilis* G_8_	From soil in greenhouse (China)	TSB-yeast extract (TSB-YE)	Static system – 25 ml culture in a 50 ml vial at 30°C	SPME – CAR/DVB/PDMS, PDMS and PDMS/DVB after reaching an OD_600_ of 1.0–1.5	Medium comparison – NIST 05 library	Liu et al. (2008a)
*B. subtilis* BL02	Rhizosphere soil of cucumber	LB	At 30°C	Method not mentioned – after 24 h	NIST 05 library	Liu et al. (2008b)
*B. subtilis* PPCB001	Surface of Valencia and Shamouti oranges (Obagwu and Korsten, 2003)	NA	Dynamic system (Purge and Trap system) – 150 ml culture	Trapping – multi-channel open tubular silicone rubber traps (MCTs)	Wiley spectral library (>80% similarity), calculation and comparison of retention times (not clear whether authentic standards have been used)	Arrebola et al. (2010)
*B. subtilis* G-1	Not defined	NA	Culture supernatant was extracted and separated using TLC	Direct GC-injection of one TLC fraction	NIST Version. 2.0 (2005)	Shifa et al. (2015)
*B. subtilis* SV75-1, SV44-2, SV36-2	From different cooked food sources (meat and vegetables)	Plate count agar (PCA, Oxoid)	Static system – 5 ml of PCA in a 20 ml vial at 28°C	SPME – CAR/PDMS at day 5 of cultivation	Medium comparison – NIST 98/Wiley ver. 6 Mass Spectral Database (probability set at >90%). Std. when possible	Chaves-López et al. (2015)
*B. subtilis* 8B-1	Potato field	LB	Static system – 100 ml in a 200 ml Erlenmeyer at 24°C	SPME – PDMS/DVB at day 5 of cultivation	NIST MS database Version 2.0 (<850)	Khabbaz et al. (2015)
*B. subtilis* XF-1	Rhizosphere soil of Chinese cabbage (*Brassica pekinensis*) (Guo et al., 2013)	LB	Static system – 7 ml in a 20 ml vial at 37°C	SPME – DVB/CAR/PDMS at 24 h of cultivation	Medium comparison – NIST/EPA/NIH Mass Spectral Database (NIST 05)	Li et al. (2015)
*B. subtilis* M29	From vermicompost	LB	Static system – cultivation in a 500 ml vial at 30°C	SPME – CAR/PDMS	NIST/MAINLIB/WileyRegistry/Rtlpest library – authentic standards whenever possible	Mu et al. (2017)
*B. subtilis* SYST2	Not-defined	MS	Static system – 30 ml of MS agar medium in a 100 ml vial at 28°C	SPME – DVB/CAR/PDMS at day 5 of cultivation	Medium comparison – NIST/EPA/NIH Mass Spectrum Library – Std. (albuterol)	Tahir et al. (2017)
*B. subtilis* FA26	Rhizosphere of rice grown in Pakistan	LB	Static system – 30 ml of MS agar medium in a 100 ml vial at 28°C	SPME – DVB/CAR/PDMS at day 3 of cultivation	Medium subtraction – NIST 11 Mass Spectrum Library (NIST11/2011/EPA/NIH) in NIST MS Search software version 2.0g (2011–05)	Rajer et al. (2017)
*B. subtilis* NCTC 10073	Not defined	Agar medium	Dynamic closed air stream system – 5 ml of MS agar medium in a 30 ml vial at 30°C	Tenax^TM^-Carbograph desorption tube – monitoring for 3 days (intervals of 24 h at 24 h, 48 h and 72 h of cultivation)	Medium subtraction – NIST MS database, version 2.0	Ratiu et al. (2017a)
*B. subtilis* CF-3	Fermented bean curd, China (Gao et al., 2016)	LB	Static system – 100 ml of MS medium in a 100 ml vial at 28°C	SPME – PA 100-μm PDMS, 7-μm PDMS from 12 h to 96 h every 12 h	Medium comparison – National Institute for Standards and Technology (NIST, 2008) Mass Spectrometry Library	Gao et al. (2018)
*B. subtilis*	Pig (*Sus scrofa domesticus*) decomposition studies	NA	Static system – 4 ml of medium in a 20 ml vial at 24°C	SPME – CWR/PDMS Arrows (in-house production) every 24 h over a 5-day period	Medium comparison – National Institute of Standards and Technology (NIST) 2014 Mass Spectral Library with a forward match factor threshold of 700 and reverse match factor threshold of 700	Eckert et al. (2018)
*B. subtilis* (MTCC 8133, 8114, and 2274)	Not defined	NA	Static system – NA plate at 28 ± 2°C	SPME – CAR/DVB at day 4 of cultivation	WILEY and NIST Library	Jangir et al. (2018)

*EPA, Environmental Protection Agency; NIH, National Institute of Health database; NIST, National Institute of Standards and Technology; TLC, thin-layer chromatography; DVB, divinylbenzene; PDMS, polydimethylsiloxane; CAR, carboxen; PA, polyacrylate; CWR, carbon wide range.*

### Methods Used for Collection, Analysis, and Compound Identification

All volatile secondary metabolites have been collected in *in vitro* systems from cultivated *B. subtilis* strains. Noteworthy is the diversity in culture conditions of bacteria, and collection techniques for volatile metabolites. These parameters should be necessarily documented. The evaluation and interpretation of potential production and emission of bacterial metabolites in nature should always be done in close consideration of culture conditions (nutrients, temperature, oxygen availability), since they determine bacterial growth and development, which of course could influence the production of volatile secondary metabolites as it was already shown for other bacterial species (Kai et al., 2009; Blom et al., 2011; Weise et al., 2012; Ratiu et al., 2017b). Furthermore, methods to collect volatile secondary metabolites can influence the classes of volatiles that are detected. Although proper descriptions of cultivation and analysis condition are sometimes missing, the following chapters will summarize the relevant parameters.

#### 1 – Culture Conditions During Collection of Volatile Secondary Metabolites

*Bacillus subtilis* isolates have mostly been cultivated on complex medium for volatile collection, including lysogeny broth (LB, often mistakenly referred to as Luria Bertani) used in seven studies and nutrient agar (NA) used in five studies (Table 1). One collection was performed using beef extract peptone broth (BEPB) and another using plate count agar (PCA, Oxoid). Murashige–Skoog medium (MS) was applied in two studies, whereby in one of these studies sucrose and tryptic soya agar (TSA) were added. Finally, in two investigations tryptic soy broth (TSB) was used. In one of these studies the authors added yeast extract (Liu et al., 2008a).

In 14 out of 19 studies, *B. subtilis* was cultivated in liquid medium during collection of volatile secondary metabolites, while in the remaining five investigations, *B. subtilis* grew on agar plates. For other bacterial species, crucial differences between cultivation on solid or in liquid medium were already demonstrated (Kai et al., 2007, 2010) most likely due to different cell numbers and varying diffusion of oxygen into the medium (Somerville and Proctor, 2013). Also, the cultivation temperature varied in the experiments in the range of 24°C and 37°C. Furthermore, collections of volatile metabolites have been performed at different time points ranging from 24 h up to 6 days of cultivation. These differences in the time of sampling play also an important role, since they reflect differences in cell numbers and developmental stages of bacterial cultures (Kai et al., 2010).

#### 2 – Collection of Volatile Secondary Metabolites

The collection of volatiles was almost exclusively performed using static or dynamic headspace systems combined with gas chromatography/electron-ionization-mass spectrometry (GC/EI-MS) (Table 1). Only in one case the bacterial filtrate was extracted and separated applying thin-layer chromatography (TLC). TLC fractions were directly injected into the GC/EI-MS system (Shifa et al., 2015). Since this procedure does not ensure that only bacterial volatiles are captured, static and dynamic headspace systems are the preferred experimental setups (Schulz and Dickschat, 2007).

##### Static headspace systems

Due to their simple application, static systems are the most favored experimental systems for volatile collection. They were used in 13 out of 19 studies. Liquid medium was inoculated with the respective *B. subtilis* isolate and filled into a cultivation vessel. This vessel was often sealed with Parafilm^®^ to avoid loss of volatiles during incubation. To extract volatile secondary metabolites from the headspace of the culture, solid phase micro extraction (SPME) was performed. A SPME fiber coated with different adsorbent materials was inserted into the headspace at defined time points of incubation. Coating materials included divinylbenzene (DVB), polydimethylsiloxane (PDMS), carboxen (CAR), polyacrylate (PA), and carbon wide range (CWR). These coatings have been used solely or in combination, e.g., the combination of CAR/DVB/PDMS (Table 1). PDMS extracts non-polar volatiles, while polyacrylate and PDMS/DVB are used to adsorb polar volatile compounds. Captured volatile secondary metabolites were thermally desorbed into the injector of the GC/EI-MS.

##### Dynamic headspace systems

The sampling of volatile secondary metabolites by streaming air over a bacterial culture changes the collection system towards a dynamic headspace system. The air was purified from pollutants and bacterial contaminations by charcoal and sterile filters, respectively. The analyzed *B. subtilis* culture grew on a Petri dish, which was placed in an analysis chamber (Ryu et al., 2004; Kai et al., 2007) or it grew in liquid medium in flasks (Arrebola et al., 2010; Ratiu et al., 2017a). After passing the bacterial culture, volatile secondary metabolites were trapped by an adsorbent material (SuperQ^®^, silicone, Tenax^®^ and others) and thereby enriched over a certain time interval. The volatiles were solvent-based or thermally desorbed and analyzed by GC/EI-MS (Table 1).

##### Identification of volatile secondary metabolites

In all presented investigations, volatile secondary metabolites have been analyzed using GC/EI-MS. In order to verify the emission by *B. subtilis* isolates, the profiles of volatiles metabolites were compared with controls, where only medium was analyzed. Unfortunately, this important control was not always properly described in several studies. The identification of volatiles mainly based on comparison of mass spectra of the analyzed compounds with corresponding mass spectra of different mass spectral libraries, for example, various versions of the Wiley library and the National Institute of Standards and Technology (NIST) library (Table 1). Among the studies considered in this review, only Robacker et al. (1998) and Ryu et al. (2004) used authentic standards and retention indices for compound identification. Other research groups only occasionally identified single volatile compounds using authentic standards, while the remaining compounds were identified only by comparison with mass spectral databases (Tahir et al., 2017). In some cases it is not recognizable if volatiles were identified using authentic standards and/or libraries (Chaves-López et al., 2015). A clear assignment of an authentic standard confirmation was unfortunately missing. Most of the authors identified merely on the basis of database suggestions using different probabilities (from 80 to 90%, Table 1). Nevertheless, for the sake of completeness, all volatile secondary metabolites that were described to be produced by *B. subtilis* were included in this review.

### Chemical Classification of Volatile Secondary Metabolites Emitted by *B. subtilis* Isolates

**FIGURE 1:**
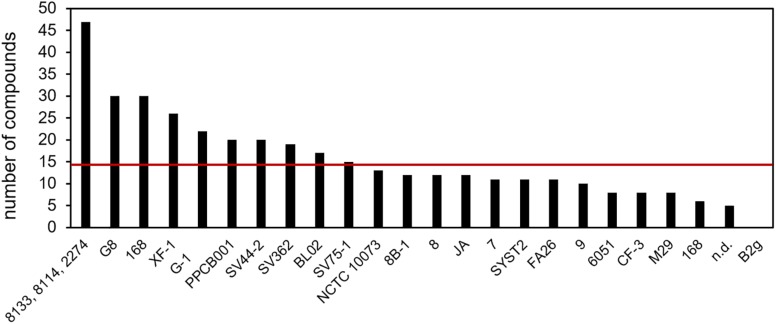
Detected compounds per *B. subtilis* isolate. Volatile secondary metabolites emitted by 26 *B. subtilis* isolates were summarized from 20 original scientific publication (see Table 1). n.d., isolate was not defined; red line is the arithmetic average of 14; *B. subtilis* 168 was analyzed twice; the results of 8133, 8114, and 2274 are aggregated in the original study.

The individual *B. subtilis* isolates emitted from 5 up to 30 compounds with an average of 14 compounds per strain (Figure 1). Surprisingly, no volatile was detected in the headspace of isolate B2g (Kai et al., 2007). One study delivered only summarized information on the volatile emission of three strains, any information on individual emission was missing (Jangir et al., 2018).

**FIGURE 2:**
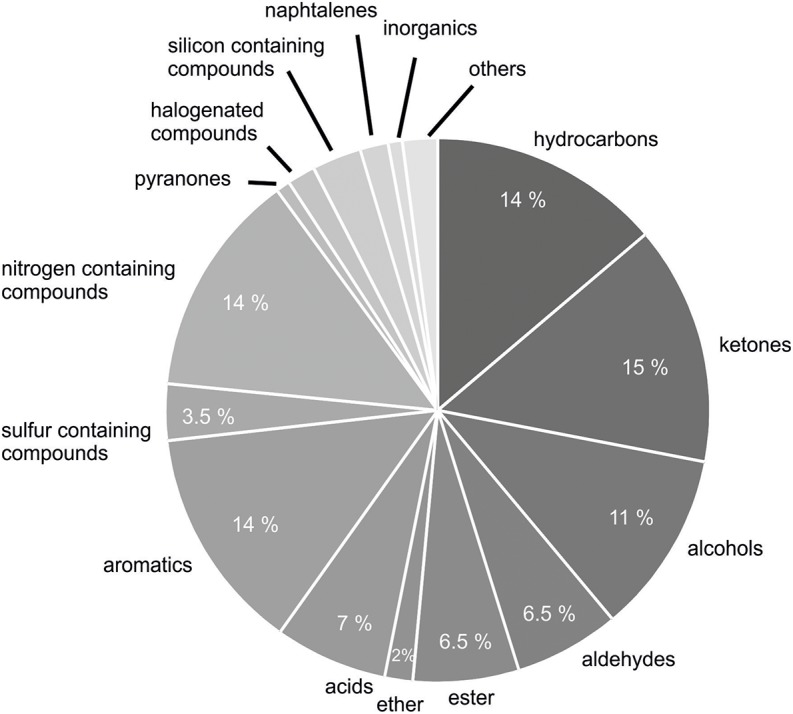
Classification of detected VOCs according to chemical classes. Two hundred and thirty-one volatile secondary metabolites emitted from 26 *B. subtilis* isolates were summarized from 20 original scientific publication (see Table 1) and grouped in chemical classes.

In total, 231 different volatile secondary metabolites were found in 26 *B. subtilis* isolates analyzed so far (Table 2). Volatile secondary metabolites of strains, which were found in the literature and yet have not been implemented in mVOC-database (Lemfack et al., 2018), have been submitted by me to the respective database in the meantime. Recently, bacterial volatile secondary metabolites were classified according to their biosynthetic origin (Schulz and Dickschat, 2007; Caulier et al., 2019). This classification was not used in this review, since the biosynthetic pathway has not been elucidated for many individual compounds. A chemical classification seemed to be more reasonable under these circumstances. The majority of detected volatile secondary metabolites were classified as ketones (34 compounds, 15%), nitrogen-containing compounds (32 compounds, 14%), hydrocarbons (33 compounds, 14%), aromatic compounds (32 compounds, 14%), and alcohols (26 compounds, 11%) (Figure 2). Furthermore, volatile secondary metabolites included aldehydes (15 compounds, 6.5%), acids (16 compounds, 7%), and esters (15 compounds, 6.5%). To a lower extent sulfur containing compounds (8 compounds, 3.5%), silicone containing compounds (7 compounds, 3%), ethers (4 compounds, 2%), halogenated compounds (2%), naphthalenes (4 compounds, 2%), pyranones (<1%), and others (5 compounds, 2%) could be detected (Figure 2). Attention has to be paid to volatiles, which are well known artifacts, e.g., silicone containing compounds (octamethyl-cyclotetrasiloxane, dodecamethyl-2-cyclohexasiloxane), 2-ethylhexanol and phthalates; however, for the sake of completeness they have also been listed (see future aspects).

**TABLE 2:** Volatile secondary metabolites emitted by *B. subtilis* isolates **a:** 8133, 8114, 2274; **b:** n.d.; **c:** CF3; **d:** NCTC 10073; **e:** M29; **f:** SYST2; **g:** FA26; **h:** SV75-1; **i:** SV44-2; **j:** SV36-2; **k:** G-1; **l:** XF-1; **m:** 168; **n:** 8B-1; **o:** PPCB001; **p:** BL02; **q:** JA, **r:** G_8_; **s:** 7; **t:** 8; **u:** 9; **v:** 168; **w:** 6051.

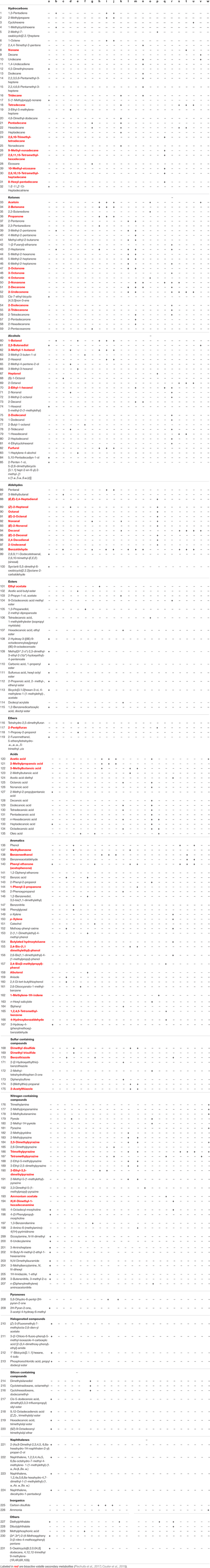

### Distribution of Volatile Secondary Metabolites Throughout the Species *B. subtilis*

**FIGURE 3:**
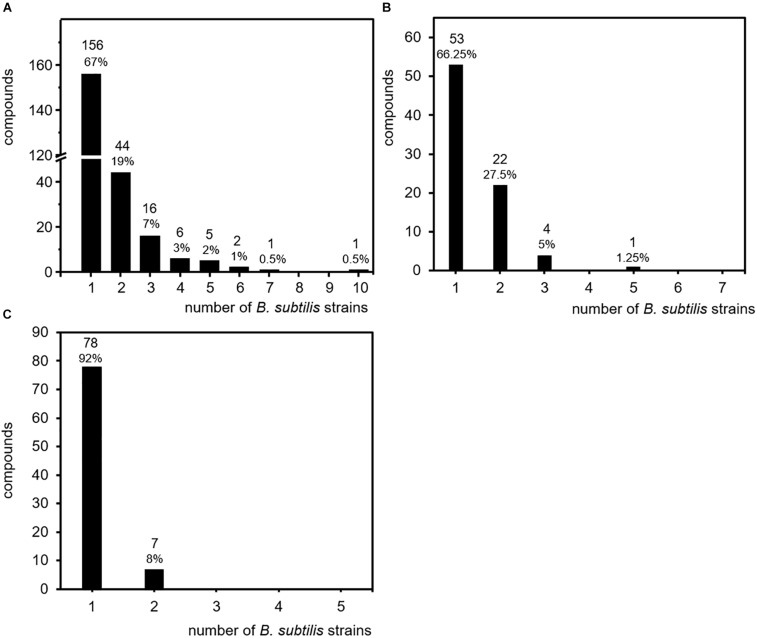
Specificity of volatile secondary metabolites. Volatile secondary metabolites emitted by 26 *B. subtilis* isolates were summarized from 20 original scientific publication (see Table 1). Compounds were related to the number of isolates responsible for emission. **(A)** Production frequency of all 231 compounds (entire data set of the survey). **(B)** Production frequency of compound emitted upon cultivation on lysogeny broth (LB). **(C)** Production frequency of compounds emitted upon cultivation on nutrient broth (NB).

In order to clarify the question of how volatile secondary metabolites are distributed within the investigated strains of *B. subtilis*, all compounds were sorted according to the number of the strains in which they were detected. Surprisingly, 156 compounds (67% of the total number of volatiles) were detected only in one single isolate, while the production of 44 compounds (19%) was observed in two isolates. Sixteen compounds (7%) were found in three isolates, six (3%) were present in four isolates, and five (2%) were detected in five isolates. Two volatiles were present in six isolates and interestingly one single compound (acetophenone) was present in seven strains and another single compound (benzaldehyde) was even present in 10 strains (Figure 3A and Table 3).

**TABLE 3:** Non-isolate-specific volatile secondary metabolites from *B. subtilis.*

**Detected in**	**Compounds**
10 isolates	Benzaldehyde
7 isolates	Phenyl ethanone (acetophenone)
6 isolates	Trimethylpyrazine, 2-Undecanone
5 isolates	Acetoin, 1-Butanol, Acetic acid, Decanal, 2-Nonanone
4 isolates	Tetradecane, Hexadecane, 2-Decanone, 3-Methyl-1-butanol, Benzeneethanol (2-phenylethanol), Diethylphthalate

These results revealed that 86% of the total amount of volatile secondary metabolites were emitted only by one or two out of 26 individual isolates of the species *B. subtilis*. These strain specific emission patterns were not expected, but may partially be explained by the diversity of culture conditions and analyses techniques. As described for other bacteria, the production and emission of secondary metabolites is influenced by nutrient supply and physico-chemical properties of the cultivation such as temperature, pH, and oxygen availability (Kai et al., 2009; Blom et al., 2011; Weise et al., 2012). Currently, there is not much data available on the nutrient dependent volatile emission of *B. subtilis*. Most investigations that are summarized here have been performed on nutrient rich media. When cultivated on LB, seven *B. subtilis* isolates released in total 80 compounds. Thereby, 53 compounds (66%) and 22 compounds (27.5%) were detected in the headspace of one or two individual *B. subtilis* isolates, respectively (Figure 3B). When cultivated on NA, five *B. subtilis* isolates released in total 85 compounds, from which 78 compounds (92%) and 7 compounds (8%) were detected in the headspace of one or two individual *B. subtilis* isolates, respectively (Figure 3C). These results resemble the overall impression of the studies. The influence of nutrient supply did not seem to be a decisive factor of the strain-specificity observed in *B. subtilis*. Although other parameters like the temperature are not yet included, these results already indicate an isolate-specific volatile emission in *B. subtilis*.

### Distribution of Biological-Active Volatile Secondary Metabolites

**FIGURE 4:**
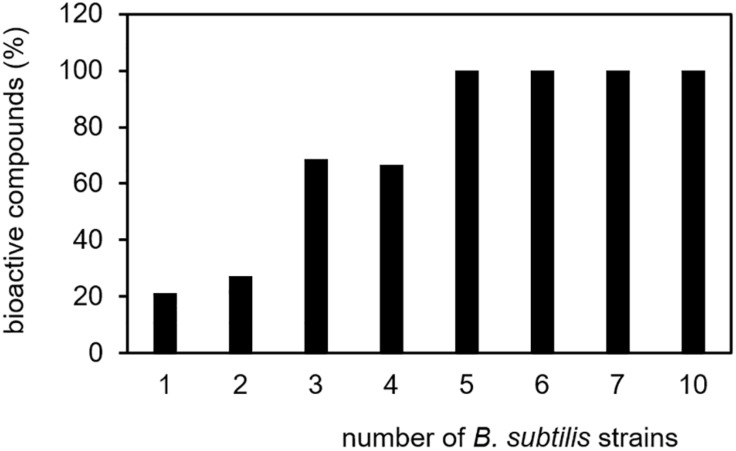
Specificity of biological-active volatile secondary metabolites. Volatile secondary metabolites emitted by 26 *B. subtilis* isolates were summarized from 20 original scientific publication (see Table 1). Biological-active compounds were related to the total number of volatiles and the number of emitting *B. subtilis* isolates.

In contrast to the isolate-specific emissions described above, the volatile metabolites benzaldehyde and acetophenone were found in several strains of *B. subtilis*. The preserved production of certain single metabolites by many strains may indicate that these compounds could provide a general advantage for *B. subtilis* in nature and it can be hypothesized that several isolates of the species maintained producing these compounds because they are bioactive. Some biological effects of volatile secondary metabolites emitted by *B. subtilis* have been already described, e.g., they can influence the growth of plants, show antimicrobial activity and affect the behavior of insects (Robacker et al., 1998; Kai et al., 2009; Caulier et al., 2019). Out of the 231 *B. subtilis* compounds listed here, 69 were recently described as bioactive (Table 2)

(Robacker et al., 1998; Piechulla et al., 2017; Caulier et al., 2019). In this list of bioactive compounds appears indeed benzaldehyde. This compound, which is known to inhibit the germination of spores and the mycelial growth of some fungi (Zou et al., 2007), was emitted by ten *B. subtilis* isolates. Also acetophenone, the single compound found in seven isolates, was described as antifungal substance and was able to directly promote plant growth (Groenhagen et al., 2013). Trimethylpyrazine and 2-undecanone, the two compounds emitted by six *B. subtilis* isolates, were described as bioactive metabolites and also all five volatiles present in the headspace of five different isolates revealed bioactivity. Of the six compounds emitted from four isolates and out of 16 compounds emitted from three isolates, four compounds (67%) and 11 compounds (69%) have bioactive properties, respectively. In contrast, only 33 compounds (21%) out of 156 compounds, which were released only by one of the isolates, have known biological activity (Figure 4). The result that various proven volatile bioactive compounds were produced and released in several isolates of the species *B. subtilis* supports the interesting conclusion that *B. subtilis* might have retained bioactive volatiles, which play a general role in communication or defense. In contrast, volatiles without a demonstrated bioactivity seemed to be strain-specifically produced. However, it has to be considered that a missing report of bioactivity does not mean that there is no bioactivity, since not all targets/activities may be known. To take this into account it can be additionally hypothesized that strain-specific production of a bioactive volatile might be an effect of special acclimation of *B. subtilis* isolates to their original habitat and thereby the volatiles might have a more specific function. Further investigations are requested in order to confirm these hypotheses. They may support the assumption that due to their biological activity, the release of specific bioactive volatile secondary metabolites is advantageous for *B. subtilis* in nature.

## Future Aspects in Volatile Secondary Metabolite Research

### Specific Emission of Volatile Secondary Metabolites: A Consequence of Acclimation or Multicellularity?

In contrast to non-volatile secondary metabolites, which are produced throughout the species *B. subtilis* (Stein, 2005), this review suggests that the emission of the majority of volatiles secondary metabolites occurs isolate-specific. This specificity might be an effect of special acclimation of the producing *B. subtilis* isolates to their (original) habitat. As the investigated strains were isolated from different sources/habitats (soil, rhizospheres, and fermented food) these isolates faced other organism from which they might have horizontally acquired genes for synthesis of volatile secondary metabolites, since the plant–soil interface is considered as hot spot of horizontal gene transfer (Heuer and Smalla, 2012; Berg et al., 2014). The transferred genes in turn might fulfill a specific biological/ecological function and thereby provide an advantage for the producing *B. subtilis* isolate to survive in the respective habitat.

Isolate-exclusive emission of volatile secondary metabolites could also be explained by multicellularity of *B. subtilis*. *B. subtilis* exhibits a variety of multicellular behaviors including different kinds of motility (swarming, swimming, etc.), sporulation, the formation of biofilms (Kalamara et al., 2018) or oligotrophic growth stage (Gray et al., 2019). This lifestyle is of advantage for *B. subtilis* due to protection from predation, increased nutrient acquisition, and enhanced resistance (Lyons and Kolter, 2015; Kalamara et al., 2018). Bacterial cells in biofilms and swarms show distinct morphologies and specific patterns and levels of gene expression and metabolic contents (Lyons and Kolter, 2015). Single cell approaches, for instance, demonstrated that genetically identical *B. subtilis* cells divide into subpopulations releasing distinct metabolic products (Rosenthal et al., 2018). The studies evaluated for this review did not contain information about heterogeneity or multicellular “stages” of the investigated strains, however, it cannot be excluded that also the heterogeneity of *B. subtilis* cultures influences the strain-specific volatile production in *B. subtilis*. In order to address this question in future research, the monitoring and analysis of volatile secondary metabolites should be related to specific stages of growth and development of *B. subtilis* cells. Single cell investigations should necessarily be performed and it is particularly interesting that especially the nutrients supply is one of the most important factors that might influence cell morphology and development (Hageman et al., 1984; Fall et al., 2006; Gallegos-Monterrosa et al., 2016).

### Which Volatile Secondary Metabolites Are Really Biosynthesized by *B. subtilis*?

The above-summarized volatiles (Table 2) have been collected from *B. subtilis* cultures. This includes bacterial cells but also the surrounding medium (solid or liquid, ingredients including peptides, sugars, organic acids, and amino acids). Compounds that originate from the medium should be excluded by proper controls. The remaining volatile compounds seem to be genuinely synthetized by the *Bacillus* cell. But it should also be considered that modification of medium-ingredients by extracellular enzymes produced by the *Bacillus* cell may account for newly appearing volatiles or that two individual volatiles may be reactants and synthesize *ex vivo* a new volatile (Kai et al., 2018). It would be very interesting to evaluate whether *B. subtilis* is able to volatilize nutrient substances in their surroundings by excretion of enzymes. In order to prove a genuine biosynthesis of volatile secondary metabolites by *Bacillus* cells, feeding experiments with selected isotopic labeled (^13^C, ^15^N) nutrient compounds (glucose, fatty acids, amino acids, or organic acids) should be performed. Isotopic labeling of certain appropriate nutrient compounds may also help to pin down volatile secondary metabolites to specific biosynthetic pathways. The biosynthesis of several microbial secondary metabolites and possible precursor substrates were already excellently described for other bacterial species (Schulz and Dickschat, 2007; Korpi et al., 2009; Scott and Piel, 2019). Nevertheless, the gap in knowledge about the biosynthesis of many volatiles in *B. subtilis* could be filled by using isotopic labeling of substrates. Knockout and overexpression mutants regarding specific steps in proposed pathways furthermore verify these results (Rijnen et al., 2003).

Labeling of nutrients will also help to exclude possible contaminants, which are also trapped and enriched during the sampling process. This applies to, e.g., silicone containing compounds and plasticizers. These compounds were found in some of the recent studies (Tahir et al., 2017; Jangir et al., 2018). They are highly artificial and their bacterial origin is very unlikely. In case of plasticizers, Dickschat already noted that it is essential to critically evaluate the source of each volatile (Dickschat, 2017). They already excluded phthalates in one of their earlier compilations (Schulz and Dickschat, 2007). Similarly, siloxanated compounds ought to be removed from the results. The presence of these compounds results mainly from Tenax^TM^-Carbotrap desorption (Ratiu et al., 2017a). Another prominent example is 2-ethylhexanol, which was present in the headspace of three *B. subtilis* isolates (168, JA and G8) (Chen et al., 2008; Liu et al., 2008a; Li et al., 2015). This compound is also considered as an artificial contaminant, although it was shown that microbial degradation of plasticizers represents also a likely source (Nalli et al., 2006).

### Emission of Volatile Secondary Metabolites From *B. subtilis* in Nature

The results summarized above were all obtained using *in vitro* test systems. In order to understand the volatile emission and their functions in nature, experimental setups are needed that simulate the habitat as adequate as possible (Kai et al., 2016). *B. subtilis* colonizes food surfaces, the soil and often occurs plant associated. Nutrient supply, physico-chemical properties of the soil and environmental factors shape both growth and development of the bacteria, but also their volatile emission (Effmert et al., 2012; Burns et al., 2015). Soil aeration and oxygen availability, for instance, are crucial for the microbial activity in soil. The oxygen supply, which *B. subtilis* experienced in the applied *in vitro* set-ups, were yet not considered, although it is conceivable that the oxygen availability for a bacterial culture on solid agar plates differs compared to culture in liquid medium (Somerville and Proctor, 2013). Another fundamental factor influencing the bacterial growth and metabolism is the temperature. Colonizing the soil, *B. subtilis* experiences changes in temperature caused by climate and season, however, in the *in vitro* tests considered in these studies, temperatures were set in the range of 24 and 37°C. Future studies should consider the factor temperature and its direct influence on the emission of *B. subtilis* volatiles in order to evaluate regional, seasonal or climate dependencies.

Evidence for nutrient dependent production of many non-volatile secondary metabolites (Tyc et al., 2017) and volatile metabolites could be yet supplied *in vitro* for several species of soil bacteria when cultivated on varying nutrient compositions (Fiddaman and Rossall, 1994; Kai et al., 2009; Blom et al., 2011; Weise et al., 2012; Ratiu et al., 2017b). One of the main nutrient resources for bacteria in soil represent plants root exudates (Barber and Martin, 1975; Lynch and Whipps, 1991; Hütsch et al., 2002). So far, the emission of volatiles by *B. subtilis* isolates was almost exclusively studied during cultivation on complex medium containing high levels of amino acids and peptides. Plants indeed release besides excess primary metabolites and among other substances amino acids and peptides via roots into the soil (Jaeger et al., 1999; Farrar et al., 2003; Uren, 2007; Dennis et al., 2010; Sasse et al., 2018). Therefore, the artificial conditions in *in vitro* studies might be close to particular natural conditions in the rhizosphere of plants, however, many other soil habitats must be considered as nutrient poor (Young et al., 2008; Schulz-Bohm et al., 2015). Therefore, isolates of *B. subtilis* should be also investigated under conditions simulating a poor soil nutrient status as well as root exudation.

It is well known that soil-born bacterial species including *B. subtilis* live in complex, diverse and dynamic communities (Fierer and Jackson, 2006; Little et al., 2008; Phelan et al., 2012). Intra- and interspecific interactions within such a microbial community can strongly affect the profiles of volatile secondary metabolites released by the community (Hol et al., 2015; Schulz-Bohm et al., 2015; Tyc et al., 2015; Schmidt et al., 2017; Kai and Piechulla, 2018; Kai et al., 2018). For instance, quorum sensing systems are assumed to affect the quality and quantity of bacterial volatiles (Kesarwani et al., 2011). Since *B. subtilis* certainly also colonizes densely populated habitats, future studies should also consider microbial interactions when investigating volatile secondary metabolite emission.

### Standards of Identification of Volatile Secondary Metabolites

The reliable and unambiguous identification of volatile secondary metabolites is the centerpiece of this research field. Schulz and Dickschat (2007) already stated that a rigorous use of the state-of-the-art methodology for compound identification could not be corroborated in all published studies. Volatile secondary metabolites are usually analyzed using GC/EI-MS. The resulting EI mass spectra are highly reproducible and show fragmentation patterns, which are characteristic for each compound. Fragmentation patterns can be submitted to databases (NIST, Whiley, etc.) resulting in a ranking of putative compounds starting with the highest score. Unequivocal identification further includes the verification of these database propositions by comparison of mass spectra and retention indices of the searched compound with the data of reference compounds (authentic standards, either commercially available or synthesized). As mentioned above, only Robacker et al. (1998) and Ryu et al. (2004) accomplished this identification workflow. Therefore, some of the compounds summarized in this review might be wrongly identified in the original paper. In future, it is recommended to follow the described workflow of identification using authentic standards. For volatile secondary metabolites, where an unambiguous identification is not possible it would be very helpful to declare them as ‘putatively identified.’ Sumner et al. (2007) suggested several confidence levels of identity for metabolomics studies (Dunn et al., 2013) (Table 4). This rating of identification should also be applicable in volatile secondary metabolite research.

**TABLE 4:** Level of identification based on Metabolomics Standard Initiative (Sumner et al., 2007, copy from Dunn et al. (2013).

**Level**	**Confidence of identity**	**Level of evidence**
1	Confidently identified compounds	Comparison of two or more orthogonal properties with an authentic chemical standard analyzed under identical analytical conditions
2	Putatively annotated compounds	Based upon physicochemical properties and/or spectral similarity with public/commercial spectral libraries, without reference to authentic chemical standards
3	Putatively annotated compound classes	Based upon characteristic physicochemical properties of a chemical class of compounds, or by spectral similarity to known compounds of a chemical class
4	Unknown compounds	Although unidentified and unclassified, these metabolites can still be differentiated and quantified based upon spectral data

### Standards of Data Acquisition

**FIGURE 5:**
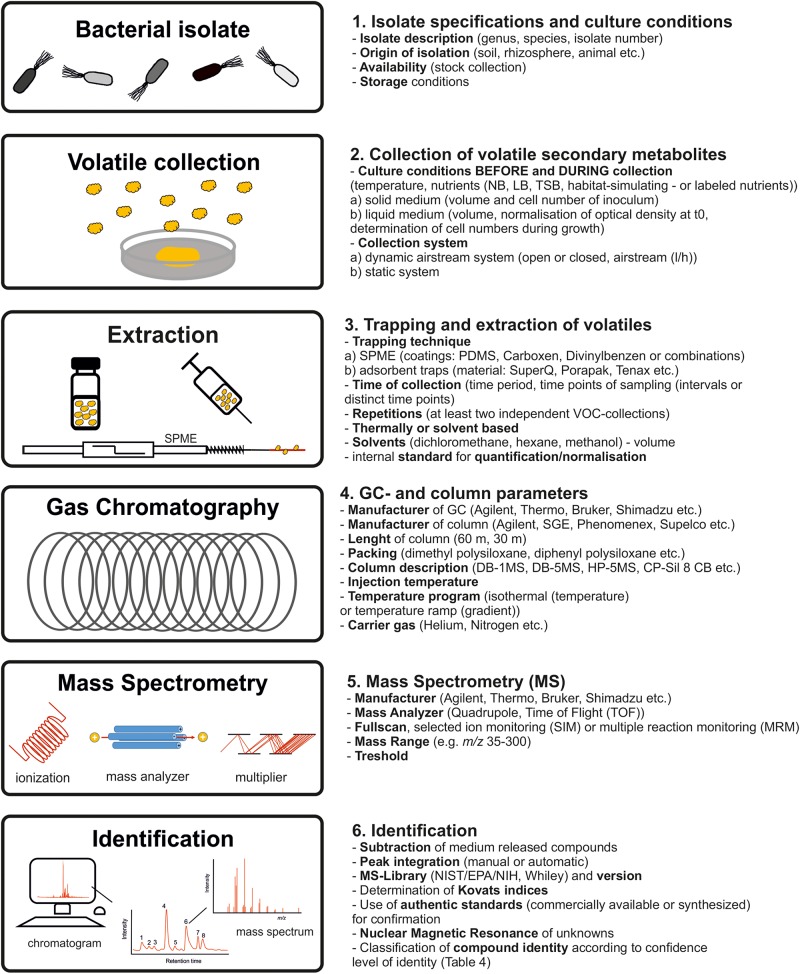
Best practice procedures and documentation. Workflow to obtain consistent, reliable and re-usable bacterial profiles in future studies.

In order to compare and summarize collected data of different studies for interpretation, and also to develop follow-up experiments, or to refine hypotheses, a clear and complete documentation is essential (Fiehn et al., 2007). Reviewing the literature on volatile secondary metabolites emitted from *B. subtilis* isolates, it became apparent that the data acquisition was often not sufficiently documented. Farag et al. (2017) described state-of-the-art procedures for volatile secondary metabolite profiling. In 2005, the metabolomics initiative was founded in order to define minimal reporting standards of metabolomics data (metabolomics standards initiative, MSI) and best practice set of reporting standards (Fiehn et al., 2007; Sumner et al., 2007). In accordance to the protocols of volatile secondary metabolite profiling and the MSI, these reporting standards should also be applied to research on bacterial volatile secondary metabolites. These suggestions encompass both the biological context and the chemical analysis (Figure 5).

The documentation should start with a comprehensive description of the *B. subtilis* isolate used as well as the origin of isolation and the storage conditions. The experimental design including cultivation parameter prior to volatile collection, during volatile collection and the used volatile collection system should be specified. The cell number of the isolates during collection should be always recorded. Methods of single time-point measurements or interval measurements during bacterial growth and the parameters of metabolite enrichment including adsorption and desorption techniques should be stated. Equally important is the description of GC-separation as well as the mass spectrometry parameters. This should also include parameter as mass scan range and thresholds. Compounds appearing in a proper control should be subtracted. The calculation of retention indices (Kováts, 1958) should facilitate comparisons with databases and other investigated isolates. For identification, in the case that suggestions of the MS-databases are used, authors should label these suggestions with a statement of similarity for each compound. It should be recognizable if volatiles were identified using standard reference compounds (synthesized or commercial) and/or libraries. The compound identity should be classified according to confidence level of identity (see above) (Sumner et al., 2007).

## Conclusion

*Bacillus subtilis* isolates emit a broad range of chemically diverse volatile secondary metabolites. About 231 volatiles have been described so far. Surprisingly, on one side some volatile secondary metabolites appeared isolate-exclusive, but on the other side other volatile secondary metabolites, which are described as bioactive molecules, were emitted more generally. The observations, however, were based on original data, which were strongly influenced by insufficient descriptions of the bacterial isolates, heterogeneous and poorly documented culture conditions as well as sampling techniques and inadequate compound identification. For a better outcome and interpretability of data as well as for the development of experimental setups or refining hypotheses, future approaches should apply well-documented workflows and fulfill state-of-the-art standards to unambiguously identify the volatile metabolites. These new approaches should question whether (I) the isolate-exclusive emission represents a consequence of acclimation or multicellularity, (II) which volatile secondary metabolites are really biosynthesized by *B. subtilis* and (III) which effect *B. subtilis* volatiles exert in nature.

## Author Contributions

MK conceived and designed the review, produced the figures and tables, and wrote the manuscript.

## Conflict of Interest

The authors declare that the research was conducted in the absence of any commercial or financial relationships that could be construed as a potential conflict of interest.
